# Cardiac failure and pulmonary hypertension secondary to renal arteriovenous malformation: a case report

**DOI:** 10.1186/s13256-021-02764-y

**Published:** 2021-03-31

**Authors:** Laura J. Albak, Ashish H. Shah, James W. Tam

**Affiliations:** 1 Internal Medicine Resident, Rady College of Medicine, Max Rady Faculty of Health Sciences, University of Manitoba, Winnipeg, Canada; 2 Section of Cardiology, Rady College of Medicine, Max Rady Faculty of Health Sciences, University of Manitoba, Winnipeg, Canada

**Keywords:** Renal arteriovenous malformation, Pulmonary hypertension

## Abstract

**Background:**

Heart failure is usually associated with a low-cardiac-output state; however, a minority of these patients are characterized by a high-output cardiac state, described as a cardiac index of > 4 L/minute/m^2^. Usually such circulation is associated with low systemic vascular resistance or arteriovenous malformation (AVM), resulting in depressurized circulation and a high-output cardiac state. Treating physicians should be cognizant of such pathology when investigating patients with heart failure. As an example, renal arteriovenous malformations are a rare vascular phenomena that are typically the result of iatrogenic, traumatic or congenital etiology. Generally, non-salient, most are detected as an incidental finding.

**Case presentation:**

A 75-year-old Afro-Caribbean man with multiple comorbidities presented to the emergency department with a 6-month history of heart failure symptoms. Cardiac catheterization demonstrated a giant right renal AVM leading to a significant left-to-right, post-tricuspid shunt that was treated with transcatheter coiling.

**Conclusions:**

We present this case to emphasize the significance of a detailed workup in a patient with heart failure symptoms.

## Introduction (background)

Heart failure is usually associated with a low-cardiac-output state; however, a minority of these patients are characterized by a high-output cardiac state, described as a cardiac index of > 4 L/minute/m^2^ [1]. Usually such circulation is associated with low systemic vascular resistance or arteriovenous malformation (AVM), resulting in depressurized circulation and a high-output cardiac state [2]. Treating physicians should be cognizant of such pathology when investigating patients with heart failure. As an example, renal AVMs are rare vascular phenomena that are typically the result of iatrogenic or traumatic events, although they can be congenital in etiology [3]. Generally non-salient and asymptomatic, most are detected as an incidental finding [4, 5]. The exception is congenital AVMs, which may create a left-to-right shunt resulting in venous congestion, pulmonary hypertension, and high-output cardiac state. A literature review (with key words “renal arteriovenous malformation” and “pulmonary hypertension”) was performed using the PubMed, MEDLINE, Embase, Cochrane, and Scopus databases. Six case reports were found [3, 6–10]. The paucity of clinical data underscores the rarity of this clinical scenario and the importance of highlighting a reversible cause of flow-related pulmonary hypertension and high-output cardiac states.

## Case presentation

A 75-year-old Afro-Caribbean man presented to the emergency department with a 6-month history of peripheral edema, dyspnea on exertion, and orthopnea. Clinical history included hypertension, gastroesophageal reflux disease, and asthma. A clinical examination revealed hypertension, oxygen saturation of 98% on room air, and hypervolemia with jugular venous distension and peripheral edema. Hematologic and biochemical investigations demonstrated normocytic normochromic anemia (hemoglobin 128 g/L) and renal insufficiency (serum creatinine 126 µmol/L).

**Fig. 1:**
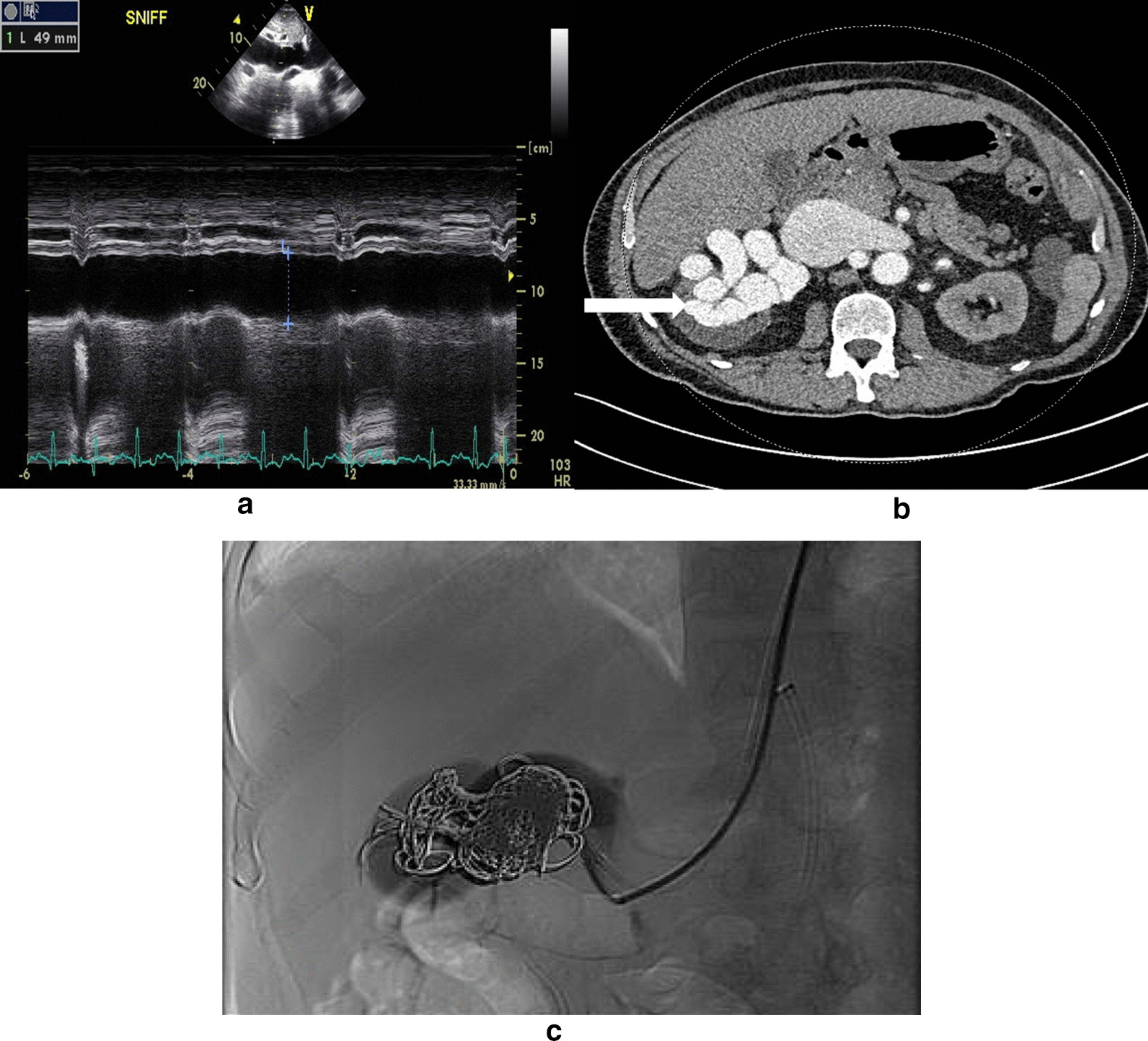
Still frame echo image of distended inferior vena cava (**a**), computed tomography of the right renal arteriovenous malformation delineated with contrast, marked by the arrow (**b**), still frame post-coil embolization catheterization (**c**)

A transthoracic echocardiogram revealed mixed systolic and diastolic left ventricular (LV) disease and an ejection fraction of 40%. The LV internal diameter in diastole was 6.0 cm. The right ventricle (RV) was moderately dilated with moderate to severe global systolic dysfunction. There was septal flattening in diastole, suggestive of volume overload with markedly elevated right atrial (RA) pressure (large inferior vena cava [IVC] in Fig. 1a). Cardiac catheterization demonstrated RA mean pressure of 19 mmHg, pulmonary artery (PA) pressure of 81/37 (mean 52) mmHg, cardiac output of 6.1 L/minute, cardiac index of 4.0 L/minute/m^2^, and increased left ventricular end-diastolic pressure with a mean capillary wedge pressure of 13 mmHg, without significant V wave. Pulmonary vascular resistance was 4 Wood units (WU) and systemic vascular resistance was 18.17 WU.

Renal ultrasound and abdominal computed tomography (CT) revealed a large complex AVM within the right renal sinus. Other investigations for alternative etiologies of pulmonary hypertension were explored and were non-contributory, including a ventilation–perfusion (V/Q) scan with low-dose CT showing no evidence of pulmonary embolism. CT angiogram displayed a large, ~ 2-cm-diameter renal AVM (Fig. 1b), which was thought to be responsible for his high-output cardiac failure. Other etiologies responsible for high-output cardiac failure were ruled out.

The patient underwent transcatheter intervention. As we wished to occlude the renal AVM beyond a branch supplying blood to the lower half of the kidney (atrophic upper half of the kidney), and we wished to avoid occluding the renal artery supplying blood to the functioning kidney, such anatomy was unsuitable for a vascular plug deployment, and hence 21 coils were deployed into the distal renal AVM (Fig. 1c). Despite partial closure of this 21-mm renal AVM, the flow through the AVM was markedly reduced, resulting in clinical improvement and a marked reduction in the IVC size. The anteroposterior dimension of the infrahepatic vena cava at the level of the superior mesenteric artery origin decreased from 4.6 to 0.5 cm. Follow-up CT angiogram demonstrated persistent but markedly reduced residual flow through the AVM.

Repeat right heart catheterization showed a reduction in RA mean pressure (10 mmHg) and PA pressure (65/33, mean 45 mmHg), with lower cardiac output (5 L/minute) and cardiac index (2.7 L/minute/m^2^). Pulmonary vascular resistance was 2.54 WU and systemic vascular resistance was 23.61 WU. The patient reported significant improvement from New York Heart Association (NYHA) class 4 to class 2 and expressed gratitude for the clinical care received.

## Discussion

High-output heart failure is a frequent presentation in emergency departments. Common etiologies include obesity, liver disease, arteriovenous shunts, lung disease, and myeloproliferative disorders [11]. Renal AVMs are a rare cause of high-output heart failure. A search of the current literature using the aforementioned criteria revealed a dearth of documented cases.

Consideration of this diagnosis early during diagnostic workup is paramount, as it can be reversible, avoiding clinical deterioration.

Renal vascular malformations are divided into congenital AVMs and arteriovenous fistulas (AVF). This is based on the communication between the arteries and veins. In congenital AVMs, there are multiple communications, creating a vascular nidus that bypasses the capillary bed. AVFs are characterized by a single direct communication between the artery and a vein, usually resulting from iatrogenic or traumatic events, or idiopathic [12]. The majority of renal AVMs are incidental findings on imaging. Symptoms may include hematuria, colicky pain, or flank fullness. Less common presentations include signs and symptoms of congestive heart failure [13].

It is prudent to investigate each of these patients very meticulously. Initial noninvasive imaging with duplex color ultrasonography reveals turbulent flow in the IVC or renal vein. Multidetector CT and contrast-enhanced magnetic resonance angiography (MRA) demonstrate dilated enhancing vessels with attenuation or signal similar to arteries. Digital subtraction angiography has the advantage of evaluating the feeding vessels and assists in planning the management of the AVMs [14].

Indications for invasive management strategies include hypertension, hematuria, AVM rupture, congestive heart failure, neoplasm, and renal failure [15]. Management options of renal AVMs include a conservative approach, transcatheter embolization, or surgery. The goal is to terminate flow through the AVM while preserving the blood supply to the renal parenchyma. Conservation management is recommended for asymptomatic AVMs. It has been shown that congenital and acquired AVMs can thrombose and resolve spontaneously.

Transcatheter embolization is the intervention of choice. If unsuccessful, or if surgical intervention is indicated, tissue-sparing is preferred to total nephrectomy [13]. In the case reported, transcatheter embolization resulted in only partial closure of the renal AVM, therefore identifying a limitation to this intervention on a reversible cause of a high-output cardiac state.

## Conclusion

Systemic AVM is a rare but potentially reversible cause of a high-output cardiac state. Clinical consideration and appropriate invasive and noninvasive workup led to salutary benefits.

## Data Availability

Not applicable.

## Abbreviations

AVM: Arteriovenous malformation
CT: Computed tomography
LV: Left ventricle
RV: Right ventricle
PA: Pulmonary artery
IVC: Inferior vena cava
AVF: Arteriovenous fistula
MRA: Magnetic resonance angiography
